# Risk Factors and Diagnosis of Diabetic Foot Ulceration in Users of the Brazilian Public Health System

**DOI:** 10.1155/2019/5319892

**Published:** 2019-09-09

**Authors:** Hígor Chagas Cardoso, Ana Laura de Sene Amâncio Zara, Suélia de Siqueira Rodrigues Fleury Rosa, Gabriel Alves Rocha, João Victor Costa Rocha, Maria Clara Emos de Araújo, Pedro de Freitas Quinzani, Yaman Paula Barbosa, Fátima Mrué

**Affiliations:** ^1^Postgraduate Program of Health Sciences, Federal University of Góias, Goiânia, Goiás 75605-050, Brazil; ^2^Postgraduate Program of Odontology, Federal University of Góias, Goiânia, Goiás 74605-220, Brazil; ^3^Postgraduate Program of Biomedical Engineering (FGA), University of Brasília, Brasília, Distrito Federal 72444-240, Brazil; ^4^Undergraduate Program of Medicine, University Center of Anápolis Unievangélica, Anápolis, Goiás 75075-010, Brazil

## Abstract

**Background:**

An individual with diabetes mellitus (DM) has an approximately 25% risk of developing ulcerations and/or destruction of the feet's soft tissues. These wounds represent approximately 20% of all causes of hospitalizations due to DM.

**Objective:**

To identify the factors for the development of diabetic foot ulceration (DFU) among individuals treated by the Brazilian public health system.

**Methods:**

This cross-sectional study was conducted on individuals with diabetes mellitus, aged above 18 years, of both sexes, and during July-October 2018 within a public healthcare unit in Brazil. All participants were assessed based on their socioeconomic, behavioral, and clinical characteristics, along with vascular and neurological evaluations. All participants were also classified according to the classification of risk of developing DFU, in accordance with the International Working Group on the Diabetic Foot (IWGDF). Statistical analyses were conducted using the chi-squared test, chi-squared test for trend, and Fisher's exact test, with a significance level of 5% (*p* < 0.05).

**Results:**

The study consisted of 85 individuals. The DFU condition was prevalent in 10.6% of the participants. Adopting the classification proposed by IWGDF, observed risks for stratification categories 0, 1, 2, and 3 were 28.2%, 29.4%, 23.5%, and 8.2%, respectively. A statistically significant (*p* < 0.05) association was observed between the development of DFU and the following variables: time since the diagnosis of diabetes and the appearance of the nails, humidity, and deformations on the feet.

**Conclusion:**

The present study found an elevated predominance of DM patients in the Brazilian public health system (SUS) featuring cutaneous alterations that may lead to ulcers; these individuals had elevated risks of developing DFU. Furthermore, it was revealed that the feet of patients were not physically examined during treatment.

## 1. Introduction

Diabetes mellitus (DM) is a metabolic disturbance resulting from constant hyperglycemia due to modifications in the production and/or the action of insulin. DM affects approximately 425 million people worldwide [1].

In 2017, Brazil, with an estimated population of 12.5 million diabetic individuals, was classified as among the 4 countries with the highest numbers of individuals with diabetes in the world. The predicted financial costs on treatment and prevention of diabetes in Brazil was found to be roughly US$ 15.7 billion in 2017 [1]. In the state of Goiás, where the current study was conducted, the expenditure on hospital treatment for diabetes amounted to US$ 700,000.00 [2].

A series of factors such as the lack awareness of the disease by both the population and health professionals, low efficiency of health services, and the undercover primary characteristics of the disease, lead to the late diagnosis of DM complications. An estimated 46.0% of occurrences in adults are not diagnosed, where 83.8% of these cases happen in developing countries [3].

The ulceration and/or destruction of soft tissue of the feet of individuals with diabetes characterize a clinical condition commonly known as the diabetic foot ulceration (DFU), responsible for 20% of all hospitalizations of people with DM [4, 5]. This condition is associated with neurological alterations and peripheral arterial disease (PAD), independent of any related infection. A person with DM is estimated to have a risk of about 25% of developing the DFU condition during his/her lifetime [4, 5]. The direct medical costs related with the treatment of DFU in Brazil was estimated to be US$ 180 million in 2014 [6].

Considering the social and economic impacts of DFU, few studies have considered the development of simpler evaluation protocols of easier applicability, aimed at the prevention and minimization of the impact of this clinical condition. These protocols are based on the evaluation of individuals diagnosed with or in high risk of developing DFU.

Thus, the current study was aimed at identifying the factors related to the development of DFU in individuals treated by the primary healthcare network in a public unit in Brazil in 2018. This paper is aimed at serving as a foundation for the further development of protocols for the diagnosis of DFU.

## 2. Materials and Methods

This cross-sectional study was conducted from July 11 to October 18, 2018, at the Dr. Ilion Fleury Jr. Health Unit, a reference unit for basic healthcare where DM cases identified in the primary healthcare network are forwarded to, within the county of Anápolis, state of Goiás, Brazil.

The probabilistic sample was estimated by means of the OpenEpi® program, version 22. To calculate the sample size, a total population of 600 patients with type 2 DM diagnosed during the 3-month study period was considered, with a prevalence of 50% of at least 1 risk for ulceration [7], an accuracy of 10%, and a confidence level of 95%. A sample of approximately 82 participants was estimated as follows:

(1) $$\begin{array}{c} n=\frac{\left[Np\left(1-p\right)\right]}{\left[\left(\left(d2/Z2\right)\left(1-\alpha \right)/2\right)*\left(N-1\right)+p*\left(1-p\right)\right]}, \end{array}$$

where *N* is the population size (in this case, 600), *p* is the expected proportion of the event (in this case, 50%), *d* is precision (in this case, 10%), *Z* is the standard score of the normal distribution (in this case, 1.96), and *α* is 5%.

During the study period, the sample size was increased to 85 patients diagnosed with type 2 DM undergoing treatment at that health facility. All patients were at least 18 years old and from both genders, needed drug treatment, and had been monitored by endocrinologists.

Five participants were excluded from the study because they had communicative or neurological disorders unrelated to DM that made it impossible for the patient to participate in the interview or limited the responses to the sensory stimuli in their feet during the clinical evaluations.

Questionnaires were used to collect data by gathering information from the Brazilian Society of Diabetes (SBD) [8] and recent publications from the Ministry of Health of Brazil [4, 5]. In the questionnaire, the variables on socioeconomic identification and characterization were collected based on the Brazil Economic Classification Criteria from the Brazilian Market Research Association (ABEP) [9], including variables related to anamnesis and previous records of the disease. General, vascular, and neurological evaluations of the patients' feet were made through physical examinations. These analyses provided the classification of the associated risk of developing DFU, a classification system validated by the International Working Group on the Diabetic Foot (IWGDF) [10] and used by the SBD, through the diagnosis of the loss of protective sensation (LOPS) and PAD.

In accordance to directives from SBD [8] and risk classification as proposed by IWGDF [10], LOPS was considered in the cases that had altered tests of 10 g monofilament associated with at least 1 altered neurological exam (vibratory sensation test using a 128 Hz tuning fork, a pinprick sensation test using a disposable pin, and/or an ankle reflex test using a tendon hammer).

The absence or reduction in size of dorsalis pedis and posterior tibial pulses associated with the Ankle Brachial Pressure Index (ABI) < 0.9 was considered as being conclusive for PAD diagnosis and used as parameters for risk classification as proposed by IWGDF [10]. ABI was quantified using a manual Doppler transducer of 8-10 MHz, by calculating systolic pressures of the arm (brachial artery) and of the ankle (anterior and posterior tibial arteries), followed by relating the greatest values of the lower limbs with the greatest value of the brachial artery [11].

Considering the IWGDF Risk Classification System, category 0 featured individuals with DM and without LOPS and PAD; category 1 featured individuals with LOPS regardless of deformities on their feet, as indicated by physical examination; category 2 considered individuals evaluated with PAD regardless of its association with LOPS; and category 3 encompassed individuals with DM where their medical history listed ulcerations or amputations [7, 10]. Individuals diagnosed with the DFU condition were those who had any type of unhealed ulceration or any destruction of soft tissue on their feet.

Data were tabulated and further analyzed using the programs Epi Info®, version 7.1 and IBM SPSS Statistics®, version 22, respectively. Absolute (*n*) and percentage (%) frequencies were used to describe categorical variables, whereas standard deviation (SD), averages, and minimum and maximum values were used to describe continuous variables. The chi-squared test for trend was used for comparison of proportions. Fisher's exact test was substituted for the chi-squared test when the expected frequency was <5 in more than 20% of the table cells and/or table cells with values <1. All statistical tests adopted a 5% (*p* < 0.05) significance level.

## 3. Ethical Considerations

This study was approved by the Research Ethics Committee of the Federal University of Goiás, with the query response number 2.504.169 on February 21, 2018.

## 4. Results and Discussion

### 4.1. Results

The sample consisted of 85 individuals whose age varied between 20 and 93 years and averaged 59.6 years (SD = ±12.8). Higher frequencies among participants were observed in the following categories: female (64.7%), married (48.2%), schooling up to elementary school diploma (37.6%), and participants in economic classes C1 (37.6%) and C2 (30.6%) (Table 1).

Regarding the medical history of patients, the mean and median diagnostic times for diabetes mellitus were 14.5 years (SD = ±9 years) and 11 years (IIQ 25-75) (8-20), respectively. Among the participants, 72.9% had relatives diagnosed with DM and 76.5% of patients used insulin regularly (Table 2). The diagnosis of PAD performed by identifying the alteration in 1 of the peripheral pulses (dorsalis pedis or tibial posterior) associated with the alteration of the ABI of 1 limb was identified in 29.4% of the participants (Table 2).

The DFU condition was prevalent for 10.6% of the participants. In the present study, based on the classification proposed by IWGDF, the observed risks for stratification category 0, 1, 2, and 3 were 28.2%, 29.4%, 23.5%, and 8.2% of the patients, respectively (Table 3). A statistically significant association was observed between the variables of medical history of previous ulcerations and the diagnosis of LOPS on the feet (*p* = 0.004) (Table 4).

The increased risk of developing DFU had statistically significant associations with the following variables: time since the diagnosis of diabetes mellitus (*p* = 0.037), altered appearance of the nails (*p* = 0.019), altered humidity (*p* = 0.029), and deformities on the feet (*p* < 0.001) (Table 5).

### 4.2. Discussion

A significant number of health professionals associate the concept of DFU exclusively with the scenario of an individual with diabetes that was further complicated due to an infection on his/her foot. This association is not correct and leads to slower diagnosis and slower adequate treatment. Existing classifications consider situations of infected wounds resulting from both neuropathy and ischemia, whereas treatments address patients with neuropathy and plantar ulcers, independent of either superficial or deep infections, and patients with critical ischemia in need of revascularization [11].

In this study, while the majority of participants were females, the average age was 59 years; these observations were made at a reference unit of basic healthcare. More women were diagnosed with DM than men, a fact that corroborates the findings of the Brazilian National Health Survey in 2013 [12], which also found that most patients with diabetes are above 60 years [12]. The higher number of older women with diabetes on treatment within specialized healthcare units may be related to the greater life expectancy of women than men, as well as the greater attention paid by women on their health. Consequently, women may be found to seek healthcare services more frequently, and thus, take preventive measures, lowering their susceptibility to significant complications such as DFU [13].

In Brazil, most (9.6%) patients diagnosed with DM had no schooling and could not complete elementary school [12]. In the current study, a higher frequency was observed among those who had an incomplete elementary schooling experience. As seen in other studies [14], the low schooling levels complicated the access to information and learning materials on diabetes, its prevention, and its complications. This fact reveals the importance and relevance of socially determined factors of diseases.

Regarding economic details, most of the individuals were in class C. The frequency of this classification is similar to that reported by Santos et al. [15]; these authors associated net income, in terms of minimum wages, with amputation rates of diabetic feet.

This research has shown that the length of time after diagnosis of DM is associated with the risk levels of developing the DFU condition. Other research studies [8, 16, 17] also established time evolution of the disease as a risk factor for developing the complications of LOPS, PAD, and DFU.

Previously diagnosed systemic arterial hypertension (SAH) has also been found to be prevalent in DM patients in this study. It must also be noted that SAH is considered a risk factor for the development of other cardiovascular diseases and for microvascular impairment, which may then lead to peripheral neuropathy, retinopathy, and nephropathy [8, 16–18].

Most participants in this study also reported treatment of dyslipidemia, which is also considered a risk factor for the development of atherosclerosis and consequently for the development of cardiovascular diseases, such as PAD [11]. Moreover, dyslipidemia is notoriously associated with peripheral neuropathy, as seen in the work of Khawaja et al. [19].

More than 80% of participants with DM in this study reported ophthalmologic alterations. The progressive weakening of vision is known as a consequence of diabetic retinopathy, which leads to limited personal care of the patient's own feet, such as incomplete feet hygiene, a limited inspection of plantar and interdigital regions, and inadequate nail cutting [4, 5, 8]. Other studies [16, 17] have shown the association between diabetic retinopathy and the development of DFU.

Among the participants, only 30% were regularly involved in physical activities, although it is commonly recommended for all patients with DM. A significant portion of participants (21.2%), higher than in other similar studies [20], was observed to have the habit of walking barefooted, contrary to medical recommendations that consider individuals with DM as more susceptible to lesions and amputations of the lower limbs [4, 5, 8].

In order to prevent further disease complications, healthcare measures for patients with diabetes, such as general informative orientations and evaluation of the feet of these patients, are fundamental and must be executed by qualified health professionals [4, 5, 8, 21]. However, only 28.2% of the patients reported any form of feet evaluation by a health professional, and only 31.8% reported receiving orientations regarding the prevention of the DFU condition. Such worrisome situations of ineffective provision and spread of preventive measures for the disease were also noticed in previous studies [8], which recommended the implementation of interdisciplinary support protocols dedicated to the early diagnosis and prevention of the DFU condition within basic healthcare units.

The physical evaluation of the patients' feet by a qualified professional is essential, since more than 10% of the participants described any sort of ulcerations of the lower limbs. The symptoms, as described by DM patients, such as “pain while walking,” “nightly pain which lowers while walking,” “muscle weakness,” “sudden twinge,” “cramps,” or “discomfort with the pressure of the blanket” may be suggestive of neuropathic and/or angiopathic alterations, which must be confirmed during neurological and vascular evaluations during physical examinations [4, 5, 7, 8, 17].

The healthcare measures to be observed by patients with diabetes included the cutting of the nails and wearing of footwear, since these observations will allow the patients to change their lifestyle accordingly, and thus prevent future complications [21–23]. In this research, only 49.4% of the participants used appropriate footwear and 44.7% properly cut their nails. In the study by Cubas et al. in [24], only 15% of the participants were observed to use appropriate footwear and 52.5% cut their nails properly. The difference between the values of the current study and that of the latter is most probably because Cubas et al. did not adhere to the current directives [5, 8], which define appropriate footwear as comfortable and closed, without hollow regions or irregular stitching.

Through physical examination, a statistically significant association was observed between the risk level for the development of DFU and the following variables: appearance of the nails, humidity, and feet deformities. Alonso-Fernández et al. [25] had also described such association (*p* < 0.01) between the identification of feet deformities and DFU.

However, the high frequency of mycotic wounds (84.7%), altered appearance of the skin (95.3%), and hyperkeratosis (94.1%) had no statistically significant association with DFU. These variables must be analyzed for DM patients, for they are considered preulcerative conditions caused by diabetic polyneuropathy and PAD [8, 10, 23].

This study identified a frequency of 29.4% of patients with conditions suggesting PAD (altered pulse palpation and ABI < 0.9), a similar value to that reported by Brito et al. [26] of 28.5%, and smaller than that of the study of Eurodiale [27], which was 50%. Conversely, these data cannot be possibly compared to those of other Brazilian studies, as most studies did not measure the ABI, although such objective procedure is recommended by the Brazilian Society of Diabetes [8], the Ministry of Health of Brazil [5], the American Diabetes Association [28], and the Society for Vascular Surgery [21]. A great number of Brazilian studies rely only on pulse palpation [14], an unreliable method of evaluation which may vary significantly depending on the observer [11]. PAD diagnosis is important for the DM patient, so as to implement the use of adequate footwear and proper medical monitoring with a vascular specialist surgeon, preventing the progression of complications of the DFU condition and its related high financial costs [29].

The examination of diabetic polyneuropathy must be initiated through the monofilament examination of a 10 g monofilament [5, 8, 21, 28], which allows the detection of loss of large fiber nerve function, related to protective plantar sensation, with an estimated sensibility of 91% [6, 30]. About 54.7% of the participants had an altered monofilament exam, according to current recommendations [5, 8, 21, 31]. However, it is not appropriate to compare the results to those of other papers that performed the same examination, since IWDGF [10] recommends the 10 g monofilament test to be undertaken on only 3 regions (1st, 3rd, and 5th metatarsal heads); however, other Brazilian studies performed the examination on 9 distinct locations of the feet [14].

To diagnose LOPS, a complementary examination of vibratory sensations (using a tuning fork of 128 Hz), pinprick sensation, and ankle reflexes, were conducted according to present directives [5, 8, 19, 28, 32]. The altered vibratory sensation, which was significantly frequent in this study (51.7%), demonstrated a statistically significant association with the risk of ulceration and amputation of the feet of DM patients in the study of Parisi et al. [17].

A stratification in risk levels after arterial and neurological examinations on DM patients is recommended, so as to correctly apply the adequate actions for each group, aiming at implementing direct preventive measures for DFU [5, 8, 32, 33]. In this study, adopting the classification proposed by IWGDF, only 28.2% of the participants were put on risk level 0, which is a frequency close to that in the study of Almeida et al. (27.8%) [18]. A particular study [14], which did not abide by the present directives of the ABI [5, 8, 21], observed a prevalence of individuals in low risk (risk level 0), varying from 56.0% to 79.8%. The situation of possible failure in the detection of DM patients which, conversely, is associated with elevated risks, may delay the qualified professional in correctly addressing the problem. It may, thus, lead to a greater risk of the patient developing ulceration or later experiencing amputation of their lower limbs.

The limitations of the current study included the use of self-reported data, which may be underestimated when concerned with the diagnosis of chronic complications, such as functions of the lack of memory, or overestimated when concerned with the practice of physical activity and foot care. This paper reports limitations related to the use of self-reported data during questionnaire administration, so they may be underestimated with regard to the diagnosis of chronic or overestimated complications regarding the practice of physical activity and foot care. Finally, a causal relationship could not be established between the dependent and independent variables due to the cross-sectional design of the research.

## 5. Conclusion

The study confirmed that the most frequent profile of DM patients during treatment, here evaluated in the Brazilian public health system, is specified by the following characteristics: females with onychocutaneous alterations precursory of ulcerations, individuals with low schooling levels, low financial conditions, and low practice of physical exercise; individuals diagnosed with DM for more than 10 years; individuals with systemic arterial hypertension, visual alterations, dyslipidemia, loss of protective sensitivity of the feet, and elevated risk of developing the DFU condition; and individuals who did not undergo the physical examination of their feet ever since the beginning of the treatment.

The risk factors that had a statistically significant association with the development of DFU were the time since the diagnosis of diabetes; appearance of the nails, humidity, and deformities on the foot; and loss of protective sensation.

## Figures and Tables

**Table 1:** Socioeconomic profiles of individuals with diabetes mellitus in a public reference healthcare unit at Anápolis, Goiás, Brazil, 2018.

Socioeconomic characteristics	
*Age (years)*	
Average (standard deviation)	59.6 (±12.8)
Range	20-93
*Gender*	*n (%)*
Male	30 (35.3)
Female	55 (64.7)
*Marital status*	
Single	11 (12.9)
Married/consensual union	41 (48.2)
Divorced/separated	17 (20.0)
Widowed	14 (16.5)
Ignored	2 (2.4)
*Schooling*	
No schooling/incomplete elementary school	13 (15.3)
Elementary school diploma/incomplete Junior high school	32 (37.6)
Junior high school diploma/incomplete high school	10 (11.8)
High school diploma/incomplete higher education	27 (31.8)
Higher education degree	3 (3.5)
*Economic classification* ^∗^ *(average monthly household income in US$)*	
B1 (2,556)	3 (3.5)
B2 (1,326)	12 (14.1)
C1 (793)	32 (37.6)
C2 (543)	26 (30.6)
D (380)	11 (12.9)
E (380)	1 (1.2)

^∗^Source: Brazilian Market Research Association in 2014 [8].

**Table 2:** Clinical analysis of individuals with diabetes mellitus treated at a reference unit of basic healthcare at Anápolis, Goiás, Brazil, 2018.

Clinical characteristics	
Average time since diagnosis of diabetes (years) (±standard deviation)	14.5 (±9.0)
*Medical history*	*n(%)*
Records of diabetes mellitus in the family	62 (72.9)
Previous use of oral hypoglycemic agents	53 (62.4)
Regular use of insulin	65 (76.5)
Systemic arterial hypertension	64 (75.3)
Dyslipidemia	57 (67.1)
Ophthalmologic alterations	72 (84.7)
Cardiovascular alterations	28 (32.9)
Smoking	41 (48.2)
The practice of physical activity	27 (31.8)
Barefoot walking habits	18 (21.2)
Feet examined by the health agent	24 (28.2)
Received feet healthcare orientations	27 (31.8)
Signs of ulceration on the feet	10 (11.8)
Pain when walking	66 (77.6)
Muscle weakness on the feet or other lower limbs	62 (72.9)
Any other diverse symptoms on the lower limbs	77 (90.6)
*Physical evaluation*	
Adequate footwear	42 (49.4)
The appearance of the nails	65 (76.5)
Nails properly cut	38 (44.7)
Mycotic wounds on the feet	72 (84.7)
Altered skin appearance	81 (95.3)
Normal hair growth	20 (23.5)
Hyperkeratosis	80 (94.1)
Anhidrosis	66 (77.6)
Deformities on the feet	5 (5.6)
Previous amputations	10 (11.8)
Peripheral arterial disease (PAD)	25 (29.4)
Loss of protective sensation (LOPS)	50 (58.8)
Altered 10 g monofilament exam	55 (54.7)
Altered test of a 128 Hz tuning fork	44 (51.7)

**Table 3:** IWGDF risk classification in individuals with diabetes mellitus attended at a reference unit of basic healthcare at Anápolis, Goiás, Brazil, 2018.

Category	*n* (%)
0	24 (28.2)
1	25 (29.4)
2	20 (23.5)
3	7 (8.2)

**Table 4:** Association between the loss of protective sensation and previous medical history of previous ulcerations on patients with diabetes mellitus who attended a reference unit of basic healthcare at Anápolis, Goiás, Brazil, 2018.

Medical history of previous ulcerations	Loss of protective sensation		*p* value^∗^
	Yes (*n* = 50)	No (*n* = 35)	
	*n* (%)	*n* (%)	
Yes	10 (100.0)	—	**0.004**
No	40 (53.3)	35 (46.7)	

^∗^Fisher's exact test (*p* < 0.05).

**Table 5:** IWGDF risk classification for the development of diabetic foot ulceration according to the clinical characteristics of individuals diagnosed with diabetes mellitus at Anápolis, Goiás, Brazil, 2018.

Clinical characteristics	Category				DFU^∗^	*p* value
	0	1	2	3		
	*n* (%)	*n* (%)	*n* (%)	*n* (%)	*n* (%)	
*Regular use of insulin*						
Yes	13 (20.0)	21 (32.3)	17 (26.2)	6 (9.2)	8 (12.3)	0.053^§^
No	11 (55.0)	4 (20.0)	3 (15.0)	1 (5.0)	1 (5.0)	
*Smoking*						
Yes	8 (19.5)	11 (26.8)	14 (34.1)	4 (9.8)	4 (9.8)	0.172^§^
No	16 (36.4)	14 (31.8)	6 (13.6)	3 (6.8)	5 (11.4)	
*Symptoms on the lower limbs*						
Yes	22 (28.6)	22 (28.6)	17 (22.1)	7 (9.1)	9 (11.7)	0.623^§^
No	2 (25.0)	3 (37.5)	3 (37.5)	—	—	
*Time since the diagnosis of diabetes (years)*						
0-10	13 (32.5)	13 (32.5)	8 (20.0)	2 (5.0)	4 (10.0)	**0.037** ^**†**^
11-20	9 (32.1)	9 (32.1)	6 (21.4)	3 (10.7)	1 (3.6)	
21-40	2 (11.8)	3 (17.6)	6 (35.3)	2 (11.8)	4 (23.5)	
*Appearance of the nails*						
Normal	11 (55.0)	2 (10.0)	6 (30.0)	1 (5.0)	—	**0.019** ^**†**^
Altered	13 (20.0)	23 (35.4)	14 (21.5)	6 (9.2)	9 (13.8)	
*Humidity of the feet*						
Normal	5 (55.6)	3 (33.3)	1 (11.1)	—	—	**0.029** ^**†**^
Altered	19 (25.0)	22 (28.9)	19 (25.0)	7 (9.2)	9 (11.8)	
*Deformities*						
Normal	24 (30.4)	25 (31.6)	20 (25.3)	4 (5.1)	6 (7.6)	**<0.001** ^**†**^
Altered	—	—	—	3 (50.0)	3 (50.0)	

^∗^DFU: diabetic foot ulceration. ^§^Chi-squared test. ^**†**^Chi-squared test for trend. *p* values in bold indicate statistically significant differences (*p* < 0.05).

## Data Availability

The data used to support the findings of this study are available from the corresponding author upon request.
